# Successful cochlear implantation in a Susac syndrome patient

**DOI:** 10.5935/1808-8694.20120045

**Published:** 2015-10-20

**Authors:** Luiz Lavinsky, Fabiana Scarton, Michelle Lavinsky-Wolff, Joel Lavinsky, Luís Henrique Motta

**Affiliations:** ^1^ MD, PhD (Professor of the Department of Otolaryngology, School of Medicine, Universidade Federal do Rio Grande do Sul, Porto Alegre, Brazil); ^2^ MD, Otolaryngologist (Otolaryngology and Head and Neck Surgery Department of Hospital de Clínicas de Porto Alegre, Porto Alegre, Brazil); ^3^ MD, Otorhinolaryngologist. MSc - Department of otorhinolaryngology - Medical School of the Federal University of Rio Grande do Sul, Porto Alegre, Brazil; ^4^ MD, Otolaryngologist (Posgraduate Program in Medical Sciences: Surgery of Universidade Federaldo Rio Grande do Sul, Porto Alegre, Brazil); ^5^ MD, Otolaryngologist, Msc (Otolaryngology and Head and Neck Surgery Department of Hospital de Clínicas de Porto Alegre, Porto Alegre, Brazil). Otolaryngology and Head and Neck Surgery Department of Hospital de Clinicas de Porto Alegre, Porto Alegre, Brazil

**Keywords:** cochlear diseases, cochlear implants, deafness, susac syndrome

## INTRODUCTION

Susac syndrome (SS) is a rare and potentially devastating disease, consisting of a triad of encephalopathy, visual defects and hearing loss, resulting from microan-giopathy of the brain, retina and cochlea. Otolaryngologists should be familiar with Susac syndrome, since hearing loss may be the initial presenting symptom. Sensorioneural Hearing loss (SNHL) in SS usually affects low and middle frequencies suggesting that this manifestation is caused mainly by microinfarcts in the apical cochlea. Although the classical triad is pathognomonic of Susac syndrome, a high index of suspicion is necessary in the majority of cases, since most patients do not present with the complete triad at the time of onset of symptoms^1^, ^2^.

Magnetic resonance imaging (MRI) is very useful in the diagnosis of SS indicating multiple small foci of increased signal intensity in T2 in both gray and white matter. Currently, there is no therapeutic algorithm for SS^2^. Thus, treatment is empirical, but there is a certainty: early treatment, even empirical, may reduce permanent sequelae^1^.

Unfortunately, there are few reports in the literature about cochlear implantation (CI) in population. The current paper describes clinical outcomes of CI in a SS patient presenting with SNHL.

## CASE REPORT

A 29-year-old man presented with bilateral segmental visual loss which was followed by headache with migrainous features. Neurologic deterioration ensued, with vomiting, loss of consciousness and amnesia. One week later, patient complained of hearing loss and aural pressure, which was followed by neurologic motor changes and cofosis. Audiometric evaluation revealed bilateral profound SNHL. Without oral reading, the word recognition was nule at open and closed set. MRI showed multiple small foci of increased signal intensity involving the corpus cal-losum and periventricular substance in T2 and FLAIR images. Retinal fluorescein angiography showed no abnormalities. Campimetry was suggestive of binasal hemianopsy. Laboratory workup was normal. That clinical picture led to the diagnosis of SS and subsequent treatment with immunoglobulin and plasmapheresis.

After treatment, there was no improvement of SNHL. Although nausea had subsided, he remained with gait instability for eighteen months after therapy institution. Besides SNHL, the patient complained of tinnitus, which worses during imbalance episodes, and bilateral aural pressure. On Romberg test, the patient swayed to the right side. Videonistagmography showed no alterations.

As audiometric evaluation showed poor results with the patient being unable to reach intelligibility percentage and demonstrating limited responses at 250 and 1000 Hz at hearing aids tests, CI was indicated.

**Figure 1:**
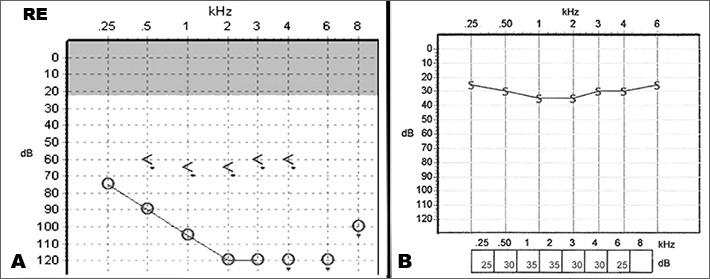
A: pre-operative right ear audiogram showing profound sensorineural hearing loss. B: 6 months postoperative audiometric evaluation with cochlear implant demonstrates threshold of 30 dB.

Patient was submitted to right CI. Facial nerve was monitored during the procedure and no trans or postoperative complications had ensued. Neural response telemetry and impedance testing results were satisfactory. After surgery, patient demonstrated good tolerance to noisy environments and was able to listen to music pleasurably. Within six months, he could talk at the telephone and showed satisfying speech recognition without oral reading. In less than a year, audiometry revealed a threshold of 30 dB and a speech recognition threshold of 100% in open field (Figure 1).

## DISCUSSION

Literature is scarce about CI results in SS patients. This report describes a successful case of a SS patient submitted to CI. Recently, Roeser et al. ^1^ published a study which reviews the otologic manifestations of 23 patients with SS and also described a unique and successful bilateral CI. Our successful results corroborate with those from Roeser et al., demonstrating that SS's patients are good candidates to CI.

## FINAL COMMENTS

Hearing loss is one of the main manifestations of SS. Literature is scarce about cochlear implants results in this population, however our paper corroborates with the few evidence available suggesting that CI can be considered as a good option for hearing rehabilitation among these patients.
